# The emergence of new prognostic scores in lung cancer patients with spinal metastasis: A 12-year single-center retrospective study

**DOI:** 10.7150/jca.60821

**Published:** 2021-07-25

**Authors:** Qing Chen, Xiaohui Chen, Lei Zhou, Fancheng Chen, Annan Hu, Ketao Wang, Haifeng Liang, Libo Jiang, Xilei Li, Jian Dong

**Affiliations:** 1 Department of Orthopeadic Surgery, Zhongshan Hospital, Fudan University, Shanghai, China.; 2 Department of Orthopaedic, First Affiliated Hospital of Xiamen University, Xiamen, 361003, Fujian, China.

**Keywords:** Lung Cancer, Spinal Metastasis, Nomogram

## Abstract

**Objective:** Lung cancer patients exhibit spinal metastases from a specific population, and with this study, we aimed to develop a model that can predict this particular group's survival.

**Methods:** Data were retrospectively collected from 83 lung cancer patients who underwent spinal metastasis surgery at our center from 2009 to 2021. After the initial assessment of treatment and scoring effects, a nomogram for survival prediction was created by identifying and integrating critical prognostic factors, followed by a consistency index (C-index) to measure consistency, and finally, a subject working characteristic curve (ROC) to compare the predictive accuracy of the three existing models.

**Results:** The mean postoperative survival was 14.7 months. Surgical treatment significantly improved the VAS and Frankel scores in lung cancer patients with spinal metastases. The revised Tokuhashi score underestimated the life expectancy of these patients. Six independent prognostic factors, including age, extraspinal bone metastasis foci, visceral metastasis, Frankel score, targeted therapy, and radiotherapy, were identified and incorporated into the model. Calibration curves for 3-, 6-, and 12-month overall survival showed a good concordance between predicted and actual risk. The nomogram C-index for the cohort study was 0.800 (95% confidence interval [CI]: 0.757-0.843). Model comparisons showed that the nomogram's prediction accuracy was better than revised Tokuhashi and Bauer's scoring systems.

**Conclusions:** Spine surgery offered patients the possibility of regaining neurological function. Having identified shortcomings in existing scoring systems, we have recreated and validated a new nomogram that can be used to predict survival outcomes in patients with spinal metastases from lung cancer, thereby assisting spinal surgeons in making surgical decisions and personalizing treatment for these patients.

## Introduction

Worldwide, lung cancer is a leading cause of cancer incidence and death^1^. Lung cancer also has the highest cancer incidence and death rate in China^2^. In the advanced stage of lung cancer, most patients develop distant metastasis, such as bone metastasis^3^. The spine is the most common site of tumor bone metastasis, and studies have reported that about 30-36% of patients with primary lung cancer will develop spinal metastasis of the tumor^4^, ^5^. Spinal metastasis can lead to neurologic dysfunction and paraplegia. These complications contribute to decreased ambulatory neurological status, quality of life (QoL), and survival^6^. Metastatic spine tumors derived from lung cancer exhibit rapid progression, leading to an unfavorable prognosis. Aggressive surgical treatment including total en-bloc spondylectomy (TES) has yielded promising results in patients with lung cancer spinal metastases, especially in patients with symptoms of pain, spinal cord compression, and spinal instability^7^-^10^. Therefore, there is a need to reassess the criteria for determining prognosis in patients considering advanced disease surgery.

Current clinicians' surgical decisions are guided by the knowledge of conceptual frameworks such as NOMS (neurological, oncological, mechanical, and systems) that consider the degree of spinal cord compression, spinal instability, radiosensitivity of the tumor, and the patient's general condition. However, with limited survival, it is difficult to assess a proposed treatment's benefits versus the overall risk-benefit ratio of an invasive procedure.

In the context of contemporary multidisciplinary treatment, many new treatment modalities like neoadjuvant therapy, surgery, and stereotactic radiotherapy are involved^11^. Several scoring systems have been developed to assess the prognosis of spinal metastatic disease including Tomita score^12^, original and revised Tokuhashi scores^13^, ^14^, and original and revised Bauer scores^15^, ^16^. However, their accuracy and reliability are not well established with lung cancer spinal metastasis.

With the aim of better predicting the best surgical candidates and adapting specific treatment or palliative care for each patient, we designed to observe the effects of surgical lung cancer spinal metastases, analyze the factors affecting patient prognosis, validate the existing prognostic evaluation system, and construct a new prognostic evaluation system for lung cancer spinal metastases using nomogram.

## Materials and methods

### Study Population

A retrospective review of the electric medical record system was conducted to retrieve clinical data for all patients diagnosed with spinal metastases from lung cancer who underwent surgery between 2009 and 2021. In our case database, 880 patients developed spinal metastatic tumors from different primary tumors: breast, lung, kidney, bladder, liver, gastrointestinal, larynx, and geologic. Our series identified 90 patients with lung cancer with spinal metastases that were accurately diagnosed by clinical imaging (CT, MRI, ECT, or PET-CT) or pathological examination. All included patients were older than 18 years, had detailed medical records, known survival time, and recent follow-up. Of these, 7 patients were excluded from the study, either because of missing data or data loss during follow-up. Ultimately, 83 patients were diagnosed with spinal metastasis either during the study or at the time of diagnosis of lung cancer. This study was approved by the Ethics Committee of Zhongshan Hospital Fudan University, and written informed consent was obtained from all patients.

### Operation category

**Figure 1:**
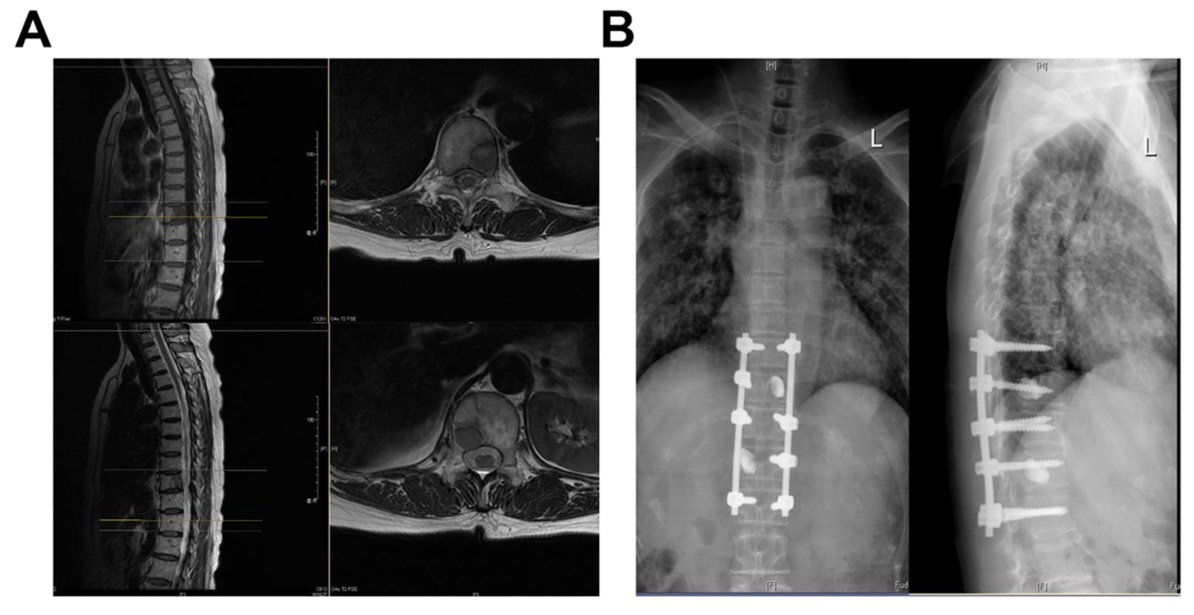
Case: Female, 60 years old, thoracic vertebrae 10 and 12 with lung cancer metastases.** (A)** Preoperative MRI; **(B)** Three Months Postoperative X-Ray.

Surgical treatment options were divided into excisional and palliative surgery. Excisional procedures include total en-bloc discectomy (TES) and intralesional excision (Case Figure 1), for example, segmental discectomy in patients with thoracolumbar metastases in a single vertebra or 2 consecutive vertebrae with an overall revised Tokuhashi score of 7-11. Palliative surgeries included: decompressive laminectomy and posterior pedicle screw insertion, mainly for patients with a revised Tokuhashi score of 0-8. The surgery was performed by experienced orthopedic surgeons from Zhongshan Hospital, Fudan University.

### Patient Characteristics

Patient Characteristics were obtained including age, gender, smoking, histological type, KPS score, visceral metastasis, extraspinal bone metastasis foci, metastases in vertebral body, Frankel score, targeted therapy, chemotherapy, radiotherapy, resection of the primary lung cancer, revised Tokuhashi score and Tomita score.

The KPS score (Table 5) was used to evaluate the general condition of patients. On a scale from 0 to 100, symptomatic patients scored 100, and patients who died scored 0. Generally, a KPS score over 80 was defined as a self-care level, 50-70 as a semi-self-care level, and 50 as a patient who needs help from others^17^.

Frankel classification provides an assessment of spinal cord function, which is classified into five grades A, B, C, D, and E according to the degree of spinal cord injury, and the grades are as follows: Grade A (complete loss of sensation and motor function below the level of injury); Grade B (no motor function, but some sensation is retained below the level of the lesion); Grade C (some muscles below the level of injury have the motor function, but no proper function). present); Grade D (proper function present below the plane of injury, walking with crutches); Grade E (typical motor and sensory function, pathological reflexes possible)^18^.

The revised Tokuhashi score (Table 6) better distinguishes between different types of primary tumors. The primary tumor area score is 0-5, while 15 points increase the other areas' score. Patients with scores above 9 will survive for 6 months, while those with scores 12-15 will survive for 12 months. The worst prognosis of 0-8 points is now adjusted to an expected survival of 6 months or less^14^.

The Tomita score (Table 7) consists of three parts, with patients in each part receiving a score of 1, 2, or 4, respectively. The essentials give the type of primary tumor, visceral metastases, and the presence of bony metastases. The total score is then converted into a survival prognosis, where the highest score (8-10) predicts survival of 3 months and the lowest score (2-4) predicts survival of 2 years. Based on the estimated overall survival, four levels of recommendation were given, three with different surgery levels and one with supportive treatment^12^.

The Bauer score (Table 8) includes the presence of pathological fractures in the decision-making process. a simplified method proposed by Leithner et al^19^ removed pathological fractures, and the Modified Bauer score generates scores in four areas, all of which can be answered yes or no to these questions. Scores for positive prognostic factors with or without visceral metastases, absence of lung cancer, primary tumor in the selected group (breast, kidney, lymphoma, multiple myeloma), and only one isolated skeletal metastasis. The total score was 0-4, where a score of 0-1 indicates a poor prognosis and no recommendation for surgery and a score of 3-4 indicates a more extensive surgery^20^.

### Statistical analyses

Different variables were used to describe the essential characteristics of the included patients. Categorical variables are characterized by frequencies and percentages, while continuous and normally distributed variables are described by means and standard deviations (SD). Univariate analysis was compared using the t-test or Wilcoxon rank-sum test for continuous variables and the chi-square test for categorical variables. The Kaplan-Meier method and between-group comparisons used the log-rank test. Multivariate cox analysis was used to test each variable's role in predicting survival outcomes, and hazard ratios (HRs) were calculated with corresponding 95% confidence intervals (CIs). Based on that, statistically significant prognostic factors were used to create a predictive nomogram of individual 3-months, 6-months, and 12-months survival. Meanwhile, the prognostic nomogram scores' predictive accuracy was evaluated using Harrell's concordance index (c-index) calculated from the function concordance index. The receiver operating characteristic (ROC) curve for censored survival data at 3-months, 6-months, and 12-months was used to test the prognostic nomogram scores^21^-^23^.

## Results

### Patient Descriptions

Overall, this study included 83 consecutive patients with a mean age of 60 years (range, 42-81 years) who underwent surgery for metastatic spinal tumors of lung cancer. Most patients (60; 72.3%) were male. Of these, 27 (32.5%) underwent excisional surgery and 56 (67.5%) underwent palliative surgery. 24 (28.9%) patients had a history of smoking. A total of 62 (74.7%) patients were diagnosed with adenocarcinoma. 38 (45.8%) patients had a good Karnofsky performance score and 45 (54.2%) had an average or poor Karnofsky performance score. Only 38 (45.8%) patients had good Karnofsky performance scores and 45 (54.2%) had moderate or poor Karnofsky performance scores. 36 (43.4%) patients had visceral metastases and 42 (50.6%) underwent postoperative radiation therapy for spinal lesions. Besides, 19 (22.9%) patients had lung cancer primary resection, 49 (59.0%) patients were receiving chemotherapy, and 36 (43.4%) patients were treated with targeted therapy. The demographic characteristics, treatment, and distribution of the patients' clinical parameters are shown in Table 1.

### Neurological Assessment

**Figure 2:**
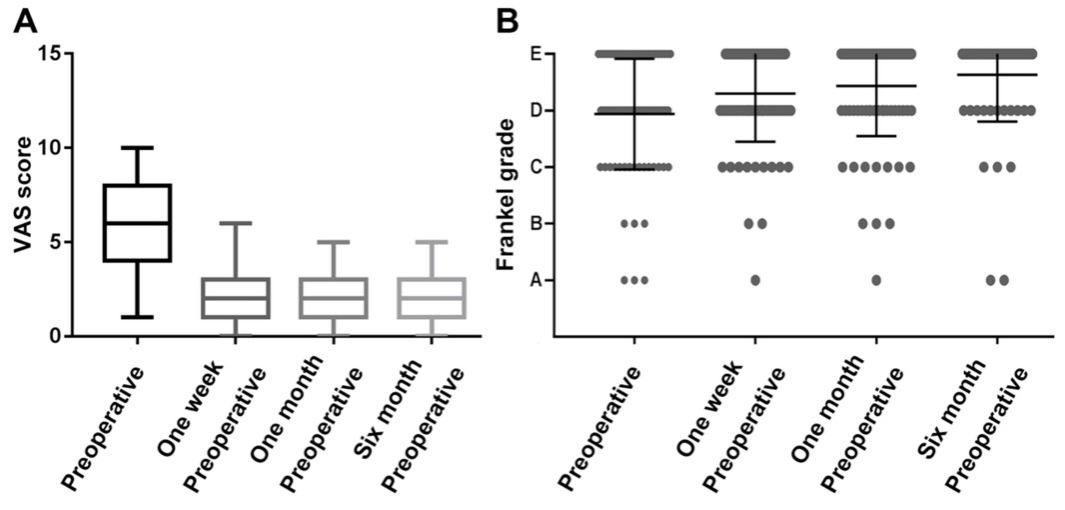
Assessment of preoperative and postoperative neurological function in patients with spinal metastases from lung cancer.** (A)** Box plot of preoperative and postoperative VAS scores; **(B)** Scattered charts of preoperative and postoperative Frankel grade.

The performance was measured using the VAS and Frankel grade classification, showing that patients had a mean VAS score of 5.6 ± 2.4 preoperatively, compared to 2.5 ± 1.4 at one week, 2.0 ± 1.2 at one month, and 2.1 ± 1.3 at six months postoperatively. The VAS was significantly decreased at all periods after surgery compared to preoperatively (Figure 2A, P<0.01). Similar results were observed in the Frankel score, with 30 (36.1%) patients improving grade 1 preoperatively than one week postoperatively. Thirty-seven patients (44.6%) improved to grade 1 and two patients improved to grade 2 in the preoperative period compared to one month after surgery. Figure 1B shows that the average preoperative Frankel grade was between C and D, and the average postoperative grade was between D and E at one week, one month, and six months after surgery. The postoperative Frankel grade was significantly improved compared with the preoperative one (Figure 2B, P<0.001).

### Survival and Tokuhashi, Tomita Score

**Figure 3:**
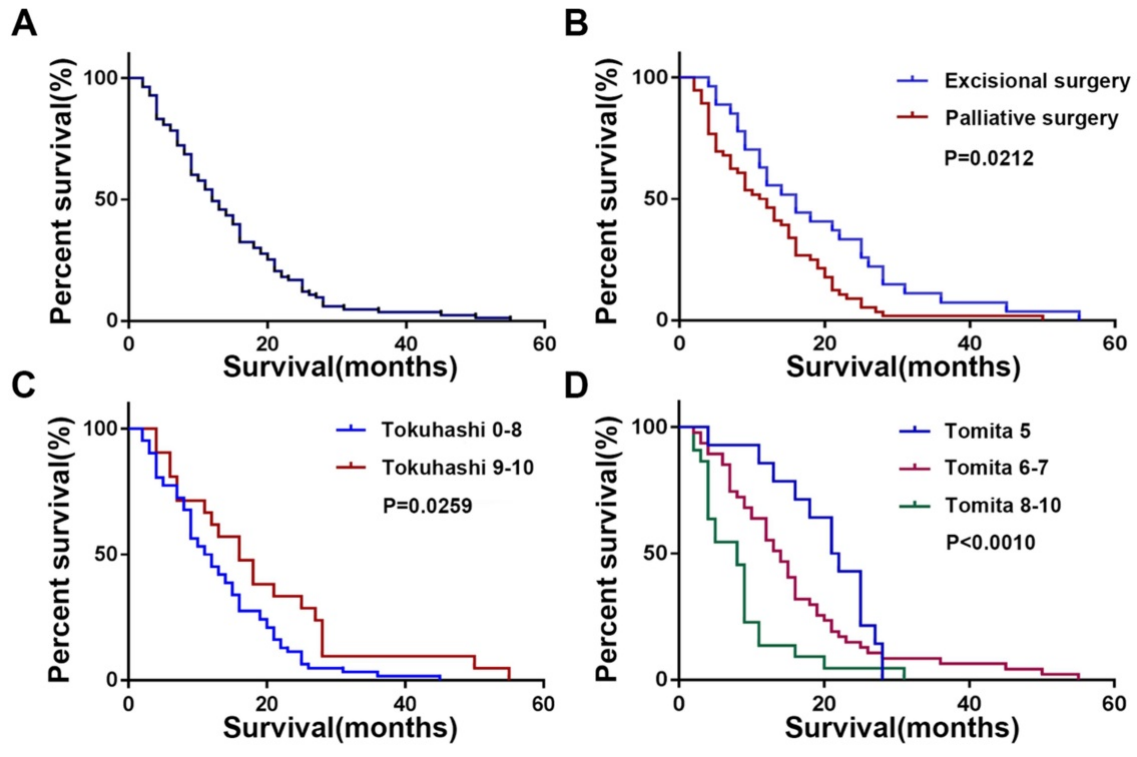
Kaplan-Meier Survival Analysis and Log-Rank Test of the patient with spinal metastasis from lung cancer.** (A)** Overall survival; **(B)** Different surgical groups;** (C)** Different Tokuhashi groups;** (D)** Different Tomita groups.

The mean postoperative survival time was 14.7 ± 10.6 months, while the median postoperative survival time was 12 months (Figure 3A). As shown in Figure 3B, patients who underwent excisional surgery (18.8±12.4 months) had significantly longer postoperative survival than those who underwent palliative surgery (12.5±8.9 months, P=0.0212). The Tomita score was divided into three groups (5, 6-7, 8-10), and there was a significant difference in postoperative survival between the three groups (Figure 3D, P<0.001). The revised Tokuhashi score was divided into groups 0-8 and 9-10, with mean postoperative survival of 13.2±8.8 months in group 0-8 and 19.0±13.5 months in group 9-10, both higher than the predictions of the modified Tokuhashi score (Figure 3C, P=0.0026), as detailed in Table 2.

### Univariate analysis and Multivariate analysis

The prognostic values of specific clinical parameters analyzed by univariate and multivariate Cox-regression analysis are shown in Tables 1 and 3. Univariate analysis showed that age, extraspinal bone metastasis foci, visceral metastasis, Frankel score, targeted therapy, and radiotherapy had a significant effect on survival. Multivariate analysis revealed that older age≥60, presence of extraspinal bone metastasis foci, and worse Frankel score were poor prognostic factors, whereas the absence of visceral metastases (P<0.001), receiving targeted therapy (P<0.001), and receiving chemotherapy (P < 0.01) were prognostically favorable.

### Development, Validation, and Prediction of Nomogram

**Figure 4:**
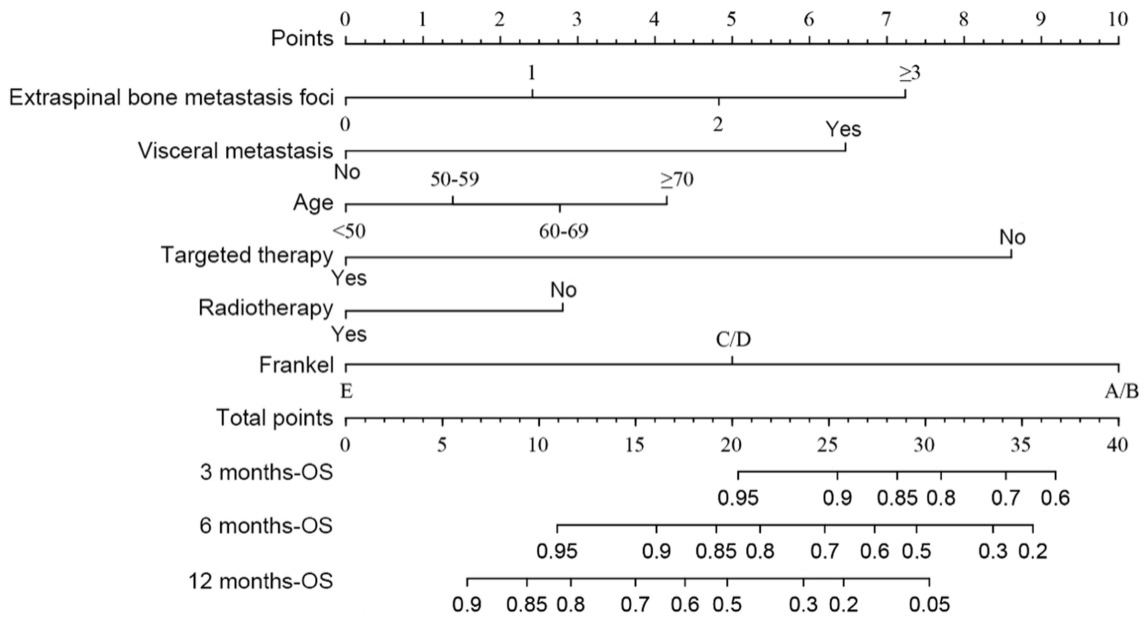
The predicting nomograms for the 3-, 6-, and 12-month survival of patients with spinal metastasis from lung cancer.

**Figure 5:**
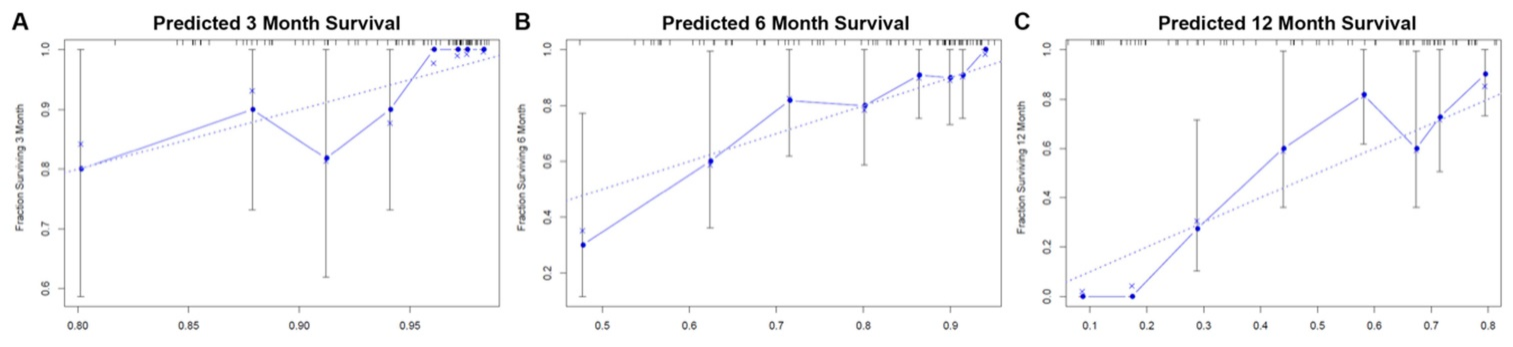
Evaluation of the nomogram for predicting survival in patients with spinal metastasis from lung cancer. **(A)** The calibration curves of 3-month survival; **(B)** The calibration curves of 6-month survival; **(C)** The calibration curves of 12-month survival.

**Figure 6:**
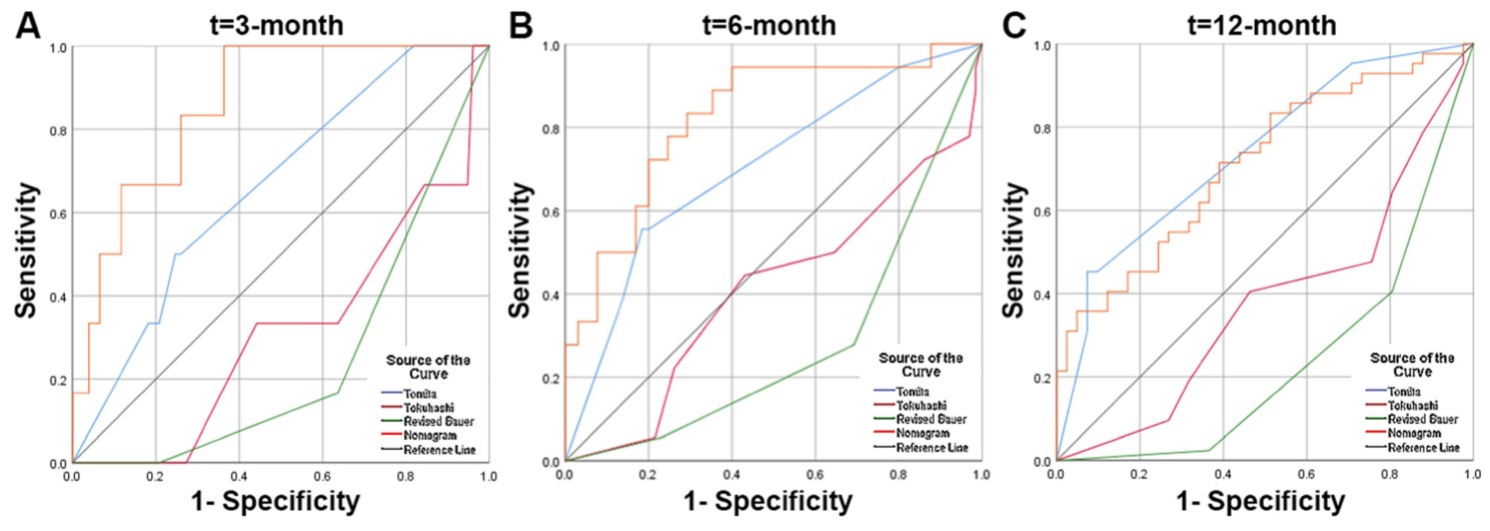
Receiver operating characteristic (ROC) curves and area under the curve (AUC) of the nomogram for predicting survival in the patients with spinal metastasis from lung cancer. **(A)** The ROC curves of 3-month survival; **(B)** The ROC curves of 6-month survival; **(C)** The ROC curves of 12-month survival.

According to the multivariate analysis prognostic factors, nomograms were constructed to predict the predicted survival at 3-months, 6-months, and 12-month postoperatively (Figure 4). The nomogram's C-index was 0.800 (95%CI, 0.757-0.843), which indicates that the nomogram exhibits a good prediction accuracy (Figure 5ABC). Further construction of the calibration curves for survival prediction at 3-month, 6-month, and 12-month postoperatively showed a good agreement between the observed probability and predicted rate (Figure 6ABC). The details can be seen in the Table 4.

## Discussion

As an aggressive disease, lung cancer spinal metastases often require surgical intervention despite recent advances in molecularly targeted therapy and immune checkpoint inhibitors^24^, ^25^. Survival prediction is associated with the decision to proceed with spine surgery. Accurate estimates of survival time help determine whether patients will benefit from surgery's expected palliative goals, which are to restore neurological function and mechanical stability to the spine while achieving pain relief^26^. There have been numerous prognostic scores and predictive models in the literature on metastatic spinal disease in past studies. Surgeons utilize prognostic scores in clinical practice to stratify patients according to the risk category and measure treatment options with this stratification. The most commonly used prognostic scores are the Tokuhashi, Tomita, Baur, Linden, Rades, and Katagiri scores, among which the revised Tokuhashi and Tomita scoring systems are widely cited^19^, ^27^. Nevertheless, most of these scoring systems are outdated and lack lung cancer specificity, such as failing to incorporate the prognostic impact of targeted therapies and hence may underestimate patients' life expectancy with lung cancer spinal metastases^28^.

In our study, the mean postoperative survival time was 14.7±10.6 months, with 72.3% of patients surviving for more than 7 months, which is similar to the findings of Hiroshi Uei et al^29^. This demonstrates that the prognosis of patients with lung cancer spinal metastases is now significantly better than in the past. Therefore, we believe that all patients with lung cancer spinal metastases should be carefully evaluated and the feasibility of surgery considered before making treatment decisions. Thanks to our hospital's multidisciplinary oncology system, the oncology, thoracic surgery, and radiotherapy departments' combined efforts have significantly improved the outcome of surgical treatment.

Surgery effectively relieves pain in patients with lung cancer spinal metastases, as more than 90% of patients in a previous study had improved VAS scores after surgery compared to preoperatively, with an average VAS score of 2.5 after surgery, which is similar to our findings. It should be pointed out that although the choice of VAS score as a criterion for pain evaluation is still subjective, it must be acknowledged that the majority of patients' pain symptoms improved effectively after surgery in both clinical and follow-up settings. Besides, 21 patients were graded Frankel A-C before surgery, and 11 of them were graded D-E after surgery. In the study by M. Lei et al^30^, 51.5% of patients with Frankel B-C grade increased to D-E after surgery, which is similar to the treatment results at our center. In summary, surgical treatment of spinal metastases from lung cancer effectively improves tumor-induced neurological impairment and reduces patient pain.

The revised Tokuhashi score is currently one of the most commonly used scoring systems to assess patients' prognosis with spinal metastases. However, in this contemporary cohort, we divided patients into 0-8 and 9-10 groups based on the revised Tokuhashi score and found that the prognosis in both groups was higher than expected with the revised Tokuhashi score, thus we believe that the revised Tokuhashi score underestimates the life expectancy of patients with spinal metastases from lung cancer. The poor performance of these scores can be attributed to demographic characteristics of the initial development and clinical therapeutic features such as targeted therapy.

Six factors were found to be independent factors associated with patient prognosis in both univariable and multivariable analyses. The importance of extraspinal bone metastasis foci and visceral metastases in predicting survival in patients who have lung cancer with spinal metastases has been widely described^12^, ^14^, ^15^. Our current data suggested that patients with lung cancer with spinal metastases have a better postoperative prognosis, so we may consider more aggressive treatment options to improve their outcomes. Given that age may change patients' treatment options and survival, we speculate that treating patients with spinal metastases from lung cancer could be more individualized to improve survival and quality of life and develop new treatment strategies. At the same time, Frankel was also included in our model. Balancing severe neurological deficits in patients with metastatic tumors, which are often predictive of poor prognosis and indicative of surgical intervention, is a clinical challenge that may be helped by the prognostic evaluation of our constructed nomogram mode^30^. It is worth noting that targeted drug therapy is currently one of the most important factors influencing the prognosis of lung cancer patients. Approximately 50% of Asian lung cancer patients are EGFR-activating mutations, and the median progression-free survival of patients with EGFR-activating mutations using EGFR-TKI is 13 months, with a median survival of 23 months l^31^, ^32^. In our study, the mean postoperative survival of patients treated with targeted agents was 19 months, significantly higher than that of patients without targeted agents, and the use of EGFR-TKI was found to improve the prognosis of patients with bone metastases from non-small cell lung cancer in a study by Sugiura et al^33^. Based on our model and experience, it is recommended that when considering the use of EGFR-TKI in patients with spinal metastases from lung cancer. Before surgery, a pathology biopsy and genetic testing should be performed, followed by consultation with the respiratory department, oncology, and other related departments to determine the further treatment plan, so that the patient can benefit more from the surgery.

In contrast to the Tomita, revised Tokuhashi, and modified Bauer scores, the constructed nomogram model had better accuracy in predicting patient survival after surgery. The nomogram model's AUC value was significantly higher than that of the revised Tokuhashi score and modified Bauer score. This was mainly because the study cohort for the revised Tokuhashi score and modified Bauer score was selected earlier than 2005, and the number of lung cancer patients included in the study cohort was relatively small. Compared with the Tomita score, the AUC values of the nomogram were higher at 3 and 6 months, and the ROC curves also showed that the nomogram model's sensitivity and specificity were higher than the Tomita score when the optimal cutoff value was selected. Thus, we believe that the nomogram model has a better predictive ability when assessing patients' survival at 3 and 6 months after surgery. The use of nomogram models can effectively predict postoperative survival time in patients with lung cancer spinal metastases, helping spinal surgeons to make precise and individualized decisions.

This research has some limitations which are inherent to retrospective studies. First, as a single-center retrospective study, a relatively small number of cases were collected, making it impossible to divide the cohort into randomized and validation groups for model construction and external validation. Secondly, our nomogram was based on patients with spinal metastases from lung cancer. It cannot be used in patients with other types of metastatic tumors. Besides, some missing variables cannot be reported, especially the lack of primary focus pathology data. Finally, the study was mainly limited to patients with spinal metastases from lung cancer who underwent surgery and may be selective in error.

## Conclusion

Surgical treatment of spinal metastases from lung cancer was effective in improving tumor-induced neurological impairment and reducing pain. After the unexpected finding, the revised Tokuhashi score underestimated patients' life expectancy with lung cancer spinal metastases. A nomogram model based on prognostic factors for predicting lung cancer patients' prognosis with spinal metastases was expected to provide a more accurate clinical decision-making tool to improve survival and patient outcomes.

## Figures and Tables

**Table 1:** Patient Demographic Characteristics, Treatment Options, and Clinical Parameters

Prognosis factors	Number (Range)	P-value
Age		0.049
Median year	60(42-81)	
<50	7(8.43%)	
50-59	30(36.14%)	
60-69	28(33.73%)	
≥70	18(21.69%)	
Gender		0.449
Male	60(72.30%)	
Female	23(27.70%)	
Smoking		0.155
Yes	24(28.90%)	
No	59(71.10%)	
Histological type		0.430
Squamous cell carcinoma	62(74.70%)	
Adenocarcinoma	9(10.80%)	
Small cell lung cancer	5(6.00%)	
Others	7(8.40%)	
KPS score		0.330
Good (80-100)	38(45.80%)	
Moderate (50-70)	34(41.00%)	
Poor (10-40)	11(13.20%)	
Visceral metastasis		0.003
Yes	36(43.40%)	
No	47(46.60%)	
Extraspinal bone metastasis foci		0.034
0	29(34.90%)	
1-2	19(22.90%)	
≥3	35(42.10%)	
Metastases in a vertebral body		0.311
Single	46(55.40%)	
Multiple	37(44.60%)	
Frankel score		0.032
E	25(30.10%)	
CD	52(62.70%)	
AB	6(7.20%)	
Targeted therapy		<0.001
Yes	36(43.40%)	
No	47(56.60%)	
Chemotherapy		0.266
Yes	49(59.00%)	
No	34(41.00%)	
Radiotherapy		0.006
Yes	42(50.60%)	
No	41(49.40%)	
Resection of the primary lung cancer		0.480
Yes	19(22.90%)	
No	64(77.10%)	

**Table 2:** Relationship Between Tomita Score, Tokuhashi Score, and Postoperative Survival in Patients

Score system	Number	Postoperative survival ≥6 months	Postoperative survival ≥12 months
Tokuhashi score			
0-8	62	48(77.4%)	31
9-10	21	19(90.5%)	14
Tomita score			
5	14	13	12
6-7	47	42	30
8-10	22	12	3

**Table 3:** Prognostic Values of Clinical Parameters According to Multivariate Cox-regression Analysis

Prognosis factors	HR (95%CI)	P-value
Age		
<50	reference	
50-59	1.023(0.834-1.560)	0.264
60-69	1.453(0.852-1.809)	0.005
≥70	1.790(1.335-2.784)	0.003
Visceral metastasis		
Yes	reference	
No	0.932(0.271-2.541)	<0.001
Extraspinal bone metastasis foci		
0	reference	
1-2	1.208(0.914-2.208)	0.003
≥3	1.534(1.214-3.291)	0.014
Frankel score		
E	reference	
CD	1.590(0.719-4.910)	0.027
AB	2.437(1.081-6.839)	<0.001
Targeted therapy		
Yes	0.264(0.256-0.291)	<0.001
No	reference	
Chemotherapy		
Yes	0.480(0.265-0.734)	0.005
No	reference	

**Table 4:** Predictive Abilities of Each Scoring System as Measured by the AUCROC at 3-, 6- and 12-Months Survival

	3-months postoperatively	6-months postoperatively	12-months postoperatively
Nomogram (SE)	0.859(0.060)	0.824(0.056)	0.713(0.056)
Tomita (SE)	0.662(0.104)	0.702(0.071)	0.736(0.054)
P	1.101	0.177	0.768
Revised Tokuhashi (SE)	0.316(0.108)	0.411(0.080)	0.380(0.062)
P	<0.001	<0.001	<0.001
Revised Bauer (SE)	0.248(0.083)	0.280(0.066)	0.235(0.052)
P	<0.001	<0.001	<0.001

**Table 5:** The KPS Score 17

Points	Point(s)
100	Normal, no complaints; no evidence of disease
90	Able to carry on normal activity; minor signs or symptoms of disease
80	Normal activity with effort; some signs or symptoms of disease
70	Cares for self; unable to carry on normal activity or to do active work
60	Requires occasional assistance, but is able to care for most of his personal needs
50	Requires considerable assistance and frequent medical care
40	Disabled; requires special care and assistance
30	Severely disabled; hospital admission is indicated although death not imminent
20	Very sick; hospital admission necessary; active supportive treatment necessary
10	Moribund; fatal processes progressing rapidly
0	Dead

**Table 6:** The Revised Tokuhashi Score

Predictive Factors	Point(s)
**General condition (Karnofsky Performance Status, %)**	
Poor (10-40)	0
Moderate (50-70)	1
Good (80-100)	2
**Number of extraspinal bone foci**	
≥3	0
1-2	1
0	2
**Number of metastasis in the vertebral body**	
≥3	0
1-2	1
0	2
**Metastasis to the major internal organs**	
Nonremovable	0
Removable	1
No metastasis	2
**Primary site of the cancer**	
Lung, osteosarcoma, stomach, bladder, esophagus, pancreas	0
Liver, gallbladder, unidentified	1
Others	2
Kidney, uterus	3
Rectum	4
Thyroid, breast, prostate, arcinoid	5
**Palsy**	
Frankel A, B (Complete)	0
Frankel C, D (Incomplete)	1
Frankel E(None)	2
**Prognostic Categories (Points)**	**Interpretation**
0-8	85% lives < 6 months with conservative treatment or palliative surgery
9-11	73% lives > 6 months (and 30% > 12 months) with palliative surgery or (exceptionally) excisional surgery
12-15	95% lives > 12 months with excisional surgery

**Table 7:** The Tomita Score

Predictive Factors	Point(s)
**Primary tumor**	
Slow growth (e.g., breast, prostate, thyroid)	1
Moderate (e.g., kidney, uterus)	2
Rapid growth (e.g., lung, liver, stomach, colon, primary unknown)	4
**Visceral metastasis**	
No visceral metastasis	0
Treatable	2
Untreatable	4
**Bone metastasis (including spine)**	
Solitary/isolated	1
Multiple	2
**Prognostic Categories (Points)**	**Interpretation**
2-3	Long-term local control (mean survival 50 months) with wide or marginal excision
4-5	Mid-term local control (mean survival 23.5 months) with marginal or intralesional excision
6-7	Short-term palliation (mean survival 15 months) with palliative surgery
8-10	Terminal care (mean survival 6months) with supportive care, no surgery

**Table 8:** The Modified Bauer Score

Predictive Factors	Point(s)
No visceral metastasis	1
No lung cancer	1
Primary tumor including breast, kidney, lymphoma, multiple myeloma	1
1 solitary skeletal metastasis	1
**Prognostic Categories (Points)**	**Interpretation**
0-1	4.8 months supportive care, no surgery
2	18.2 months short-term palliation, dorsal surgery
3-4	28.4 monthsemid-term local control, dorsoventral surgery

## Abbreviations

LC: lung cancer
CT: computed tomography
MRI: magnetic resonance imaging
PET: position emission tomography
TES: total en bloc spondylectomy
KPS: Karnofsky score
VAS: visual analog scale
HR: hazard ratio
CI: confidence interval

## References

[1] Bray F, Ferlay J, Soerjomataram I, Siegel RL, Torre LA, Jemal A (2018). Global cancer statistics 2018: GLOBOCAN estimates of incidence and mortality worldwide for 36 cancers in 185 countries. CA Cancer J Clin.

[2] Zheng RS, Sun KX, Zhang SW, Zeng HM, Zou XN, Chen R (2019). [Report of cancer epidemiology in China, 2015]. Zhonghua Zhong Liu Za Zhi.

[3] Esposito M, Mondal N, Greco TM, Wei Y, Spadazzi C, Lin SC (2019). Bone vascular niche E-selectin induces mesenchymal-epithelial transition and Wnt activation in cancer cells to promote bone metastasis. Nat Cell Biol.

[4] Hernandez RK, Wade SW, Reich A, Pirolli M, Liede A, Lyman GH (2018). Incidence of bone metastases in patients with solid tumors: analysis of oncology electronic medical records in the United States. BMC Cancer.

[5] Wong DA, Fornasier VL, MacNab I (1990). Spinal metastases: the obvious, the occult, and the impostors. Spine (Phila Pa 1976).

[6] Barzilai O, McLaughlin L, Amato MK, Reiner AS, Ogilvie SQ, Lis E (2018). Predictors of quality of life improvement after surgery for metastatic tumors of the spine: prospective cohort study. Spine J.

[7] Patchell RA, Tibbs PA, Regine WF, Payne R, Saris S, Kryscio RJ (2005). Direct decompressive surgical resection in the treatment of spinal cord compression caused by metastatic cancer: a randomised trial. Lancet.

[8] Guo C, Yan Z, Zhang J, Jiang C, Dong J, Jiang X (2011). Modified total en bloc spondylectomy in thoracic vertebra tumour. Eur Spine J.

[9] Duan PG, Li RY, Jiang YQ, Wang HR, Zhou XG, Li XL (2015). Recurrent adamantinoma in the thoracolumbar spine successfully treated by three-level total en bloc spondylectomy by a single posterior approach. Eur Spine J.

[10] Zheng GL, Zhou H, Zhou XG, Lin H, Li XL, Dong J (2018). Is Traditional Closed Thoracic Drainage Necessary to Treat Pleural Tears After Posterior Approach Thoracic Spine Surgery?. Spine (Phila Pa 1976).

[11] Chang JH, Shin JH, Yamada YJ, Mesfin A, Fehlings MG, Rhines LD (2016). Stereotactic Body Radiotherapy for Spinal Metastases: What are the Risks and How Do We Minimize Them?. Spine (Phila Pa 1976).

[12] Tomita K, Kawahara N, Kobayashi T, Yoshida A, Murakami H, Akamaru T (2001). Surgical strategy for spinal metastases. Spine (Phila Pa 1976).

[13] Tokuhashi Y, Matsuzaki H, Toriyama S, Kawano H, Ohsaka S (1990). Scoring system for the preoperative evaluation of metastatic spine tumor prognosis. Spine (Phila Pa 1976).

[14] Tokuhashi Y, Matsuzaki H, Oda H, Oshima M, Ryu J (2005). A revised scoring system for preoperative evaluation of metastatic spine tumor prognosis. Spine (Phila Pa 1976).

[15] Bauer HC, Wedin R (1995). Survival after surgery for spinal and extremity metastases. Prognostication in 241 patients. Acta Orthop Scand.

[16] Bauer H, Tomita K, Kawahara N, Abdel-Wanis ME, Murakami H (2002). Surgical strategy for spinal metastases. Spine (Phila Pa 1976).

[17] Terret C, Albrand G, Moncenix G, Droz JP (2011). Karnofsky Performance Scale (KPS) or Physical Performance Test (PPT)? That is the question. Crit Rev Oncol Hematol.

[18] van Middendorp JJ, Goss B, Urquhart S, Atresh S, Williams RP, Schuetz M (2011). Diagnosis and prognosis of traumatic spinal cord injury. Global Spine J.

[19] Leithner A, Radl R, Gruber G, Hochegger M, Leithner K, Welkerling H (2008). Predictive value of seven preoperative prognostic scoring systems for spinal metastases. Eur Spine J.

[20] Carrwik C, Olerud C, Robinson Y (2020). Predictive Scores Underestimate Survival of Patients With Metastatic Spine Disease: A Retrospective Study of 315 Patients in Sweden. Spine (Phila Pa 1976).

[21] Hao L, Pan J, Wang D, Bi YW, Ji JT, Xin L (2017). Risk factors and nomogram for pancreatic pseudocysts in chronic pancreatitis: A cohort of 1998 patients. J Gastroenterol Hepatol.

[22] Royston P, Altman DG (2013). External validation of a Cox prognostic model: principles and methods. BMC Med Res Methodol.

[23] Yang Y, Zhang YJ, Zhu Y, Cao JZ, Yuan ZY, Xu LM (2015). Prognostic nomogram for overall survival in previously untreated patients with extranodal NK/T-cell lymphoma, nasal-type: a multicenter study. Leukemia.

[24] Pruitt AA (2017). Epidemiology, Treatment, and Complications of Central Nervous System Metastases. Continuum (Minneap Minn).

[25] Park SJ, Lee CS, Chung SS (2016). Surgical results of metastatic spinal cord compression (MSCC) from non-small cell lung cancer (NSCLC): analysis of functional outcome, survival time, and complication. Spine J.

[26] Kim JM, Losina E, Bono CM, Schoenfeld AJ, Collins JE, Katz JN (2012). Clinical outcome of metastatic spinal cord compression treated with surgical excision ± radiation versus radiation therapy alone: a systematic review of literature. Spine (Phila Pa 1976).

[27] Sun JM, Ahn JS, Lee S, Kim JA, Lee J, Park YH (2011). Predictors of skeletal-related events in non-small cell lung cancer patients with bone metastases. Lung Cancer.

[28] Lei M, Liu Y, Tang C, Yang S, Liu S, Zhou S (2015). Prediction of survival prognosis after surgery in patients with symptomatic metastatic spinal cord compression from non-small cell lung cancer. BMC Cancer.

[29] Uei H, Tokuhashi Y, Maseda M (2017). Treatment Outcome of Metastatic Spine Tumor in Lung Cancer Patients: Did the Treatments Improve Their Outcomes?. Spine (Phila Pa 1976).

[30] Lei M, Liu Y, Liu S, Wang L, Zhou S, Zhou J (2016). Individual strategy for lung cancer patients with metastatic spinal cord compression. Eur J Surg Oncol.

[31] Miller VA, Riely GJ, Zakowski MF, Li AR, Patel JD, Heelan RT (2008). Molecular characteristics of bronchioloalveolar carcinoma and adenocarcinoma, bronchioloalveolar carcinoma subtype, predict response to erlotinib. J Clin Oncol.

[32] Mitsudomi T, Morita S, Yatabe Y, Negoro S, Okamoto I, Tsurutani J (2010). Gefitinib versus cisplatin plus docetaxel in patients with non-small-cell lung cancer harbouring mutations of the epidermal growth factor receptor (WJTOG3405): an open label, randomised phase 3 trial. Lancet Oncol.

[33] Sugiura H, Yamada K, Sugiura T, Hida T, Mitsudomi T (2008). Predictors of survival in patients with bone metastasis of lung cancer. Clin Orthop Relat Res.

